# Tumor vessel co-option: The past & the future

**DOI:** 10.3389/fonc.2022.965277

**Published:** 2022-08-31

**Authors:** Anne Cuypers, Anh-Co Khanh Truong, Lisa M. Becker, Paula Saavedra-García, Peter Carmeliet

**Affiliations:** ^1^ Laboratory of Angiogenesis and Vascular Metabolism, Center for Cancer Biology (CCB), Vlaams Instituut voor Biotechnologie (VIB) and Department of Oncology, Leuven Cancer Institute (LKI), KU Leuven, Leuven, Belgium; ^2^ Laboratory of Angiogenesis and Vascular Heterogeneity, Department of Biomedicine, Aarhus University, Aarhus, Denmark; ^3^ Center for Biotechnology, Khalifa University of Science and Technology, Abu Dhabi, United Arab Emirates

**Keywords:** vessel co-option, anti-angiogenic therapy resistance, tumor vascularization, mouse models, molecular mechanisms, state-of-the-art analysis

## Abstract

Tumor vessel co-option (VCO) is a non-angiogenic vascularization mechanism that is a possible cause of resistance to anti-angiogenic therapy (AAT). Multiple tumors are hypothesized to primarily rely on growth factor signaling-induced sprouting angiogenesis, which is often inhibited during AAT. During VCO however, tumors invade healthy tissues by hijacking pre-existing blood vessels of the host organ to secure their blood and nutrient supply. Although VCO has been described in the context of AAT resistance, the molecular mechanisms underlying this process and the profile and characteristics of co-opted vascular cell types (endothelial cells (ECs) and pericytes) remain poorly understood, resulting in the lack of therapeutic strategies to inhibit VCO (and to overcome AAT resistance). In the past few years, novel next-generation technologies (such as single-cell RNA sequencing) have emerged and revolutionized the way of analyzing and understanding cancer biology. While most studies utilizing single-cell RNA sequencing with focus on cancer vascularization have centered around ECs during sprouting angiogenesis, we propose that this and other novel technologies can be used in future investigations to shed light on tumor EC biology during VCO. In this review, we summarize the molecular mechanisms driving VCO known to date and introduce the models used to study this phenomenon to date. We highlight VCO studies that recently emerged using sequencing approaches and propose how these and other novel state-of-the-art methods can be used in the future to further explore ECs and other cell types in the VCO process and to identify potential vulnerabilities in tumors relying on VCO. A better understanding of VCO by using novel approaches could provide new answers to the many open questions, and thus pave the way to develop new strategies to control and target tumor vascularization.

## Introduction

Sprouting angiogenesis is often regarded as the most significant mechanism of tumor vascularization and thus became the main target for anti-angiogenic therapy (AAT) (1). However, tumors are also able to ensure their blood and nutrient supply by means of alternative vascularization mechanisms, such as vessel splitting (intussusceptive angiogenesis), vascular mimicry, and vessel co-option (VCO) (2–5). Vessel co-option has first been observed by Francesco Pezzella in his pioneering work published in the mid 90s (2, 3), where he described an “alveolar or putative nonangiogenic” vascularization pattern. Interestingly, besides the acknowledgement of alternative vascularization patterns, in the 2011 new addition of the landmark review on the “Hallmarks of cancer: The new generation” (6), tumor vascularization was still described as “induction of angiogenesis”. Only this year, Doug Hanahan acknowledged in the updated “The Hallmarks of cancer: New dimensions” publication the importance of alternative modes of tumor vascularization and updated the hallmark to “inducing or assessing vasculature” (7). Thus, besides being discovered for almost three decades, only now the importance of VCO is being realized more completely by the scientific community. It is therefore not surprising that only little is known about this complex process.

**Figure 1:**
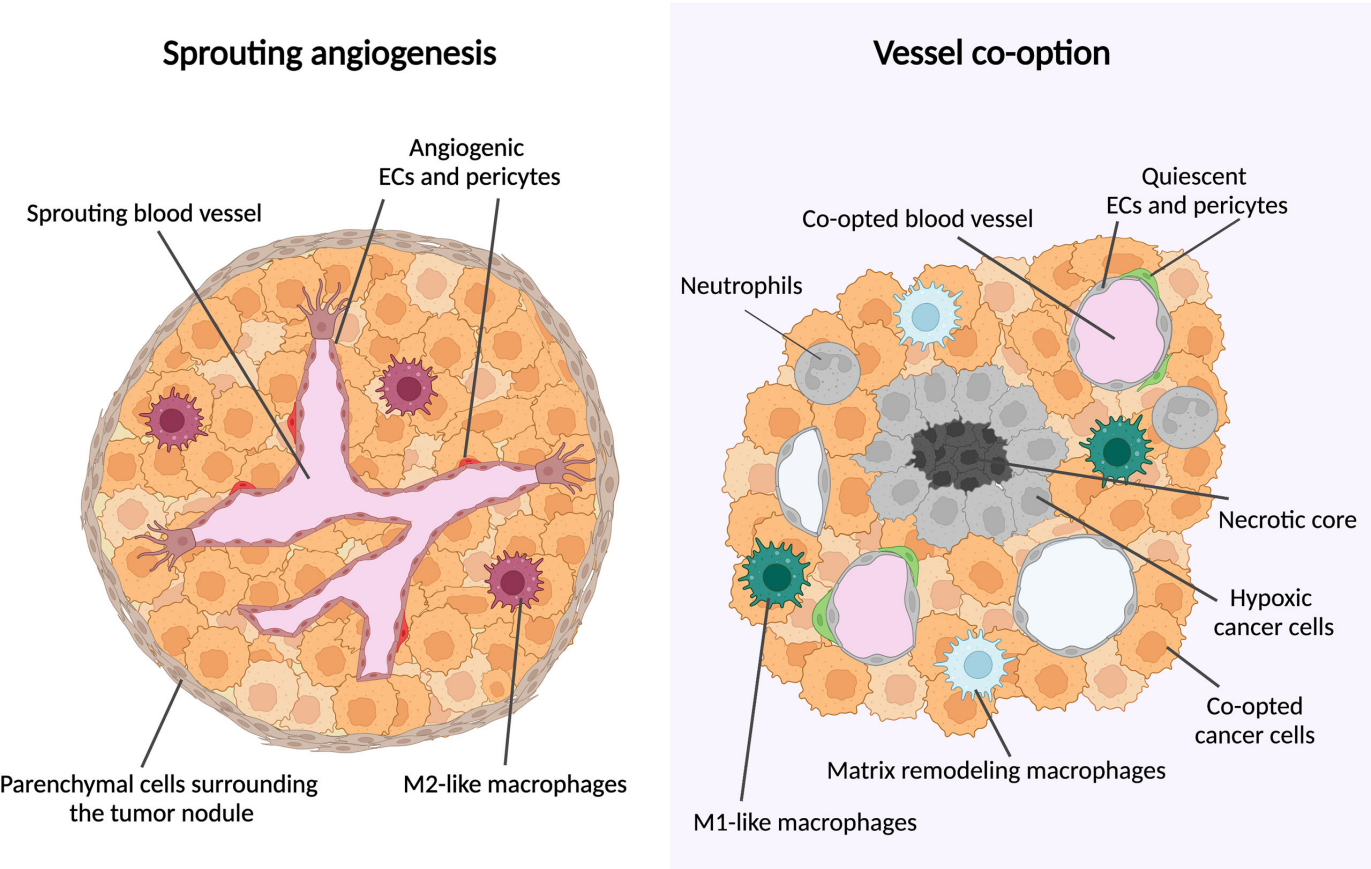
Metastatic tumor growth *via* vessel co-option versus sprouting angiogenesis. Schematic overview of metastatic tumor growth by vessel co-option versus sprouting angiogenesis. Growth *via* vessel co-option (left panel): During vessel co-option, cancer cells co-opt the healthy lung structures in an irregular and infiltrative manner, resulting in a tumor with a necrotic core. Cell types thus far associated with vessel co-option are indicated (neutrophils, M1-like macrophages, matrix-remodeling macrophages). Growth *via* sprouting angiogenesis (right panel): Metastases growing mainly *via* sprouting angiogenesis are characterized by a globular shape, excluding healthy alveolar cells. New blood vessel formation is achieved by proliferation and migration of ECs out of pre-existing blood vessels. M2-like macrophages are enriched in metastases growing *via* sprouting angiogenesis. This figure is adapted from (10).

During sprouting angiogenesis, vessel growth occurs by proliferation and migration of endothelial cells (ECs) from preexisting vessels; during VCO, there is no new blood vessel formation but instead, cancer cells hijack pre-existing blood vessels to grow and to invade the healthy tissue (8, 9) (**Figure 1**). A main feature of co-opting cancer cells is an increased motility and invasion to grow along pre-existing vessels (8, 11, 12). Often, the surrounding cancer cells compress the co-opted vessel, which can generate a hypoxic tumor core (13, 14) (**Figure 1**).

**Figure 2:**
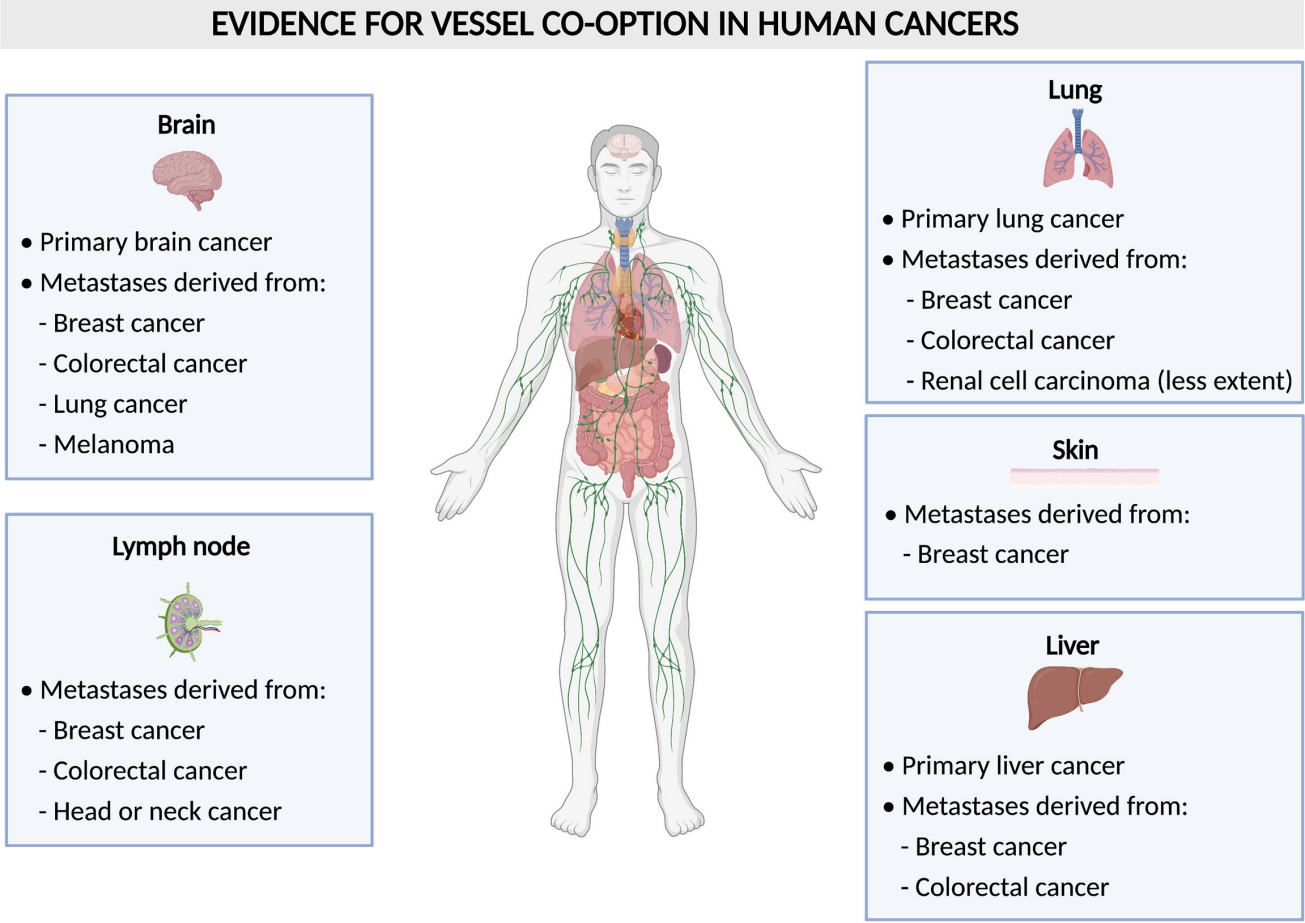
Vessel co-option in human tumors. Schematic overview of human tumors (per organ) with evidence for vessel co-option.

VCO is largely understudied when compared to sprouting angiogenesis, however, it is potentially of great interest with regards to cancer therapy, as many studies suggest VCO to present a resistance mechanism against AAT (9, 15–17). Furthermore, VCO is known to be associated with worse prognosis and is frequently occurring both in primary tumors as well as in metastases (8, 9). The most common organs, in which tumors are described to use non-angiogenic mechanisms such as VCO are the liver, lung and brain, both for primary tumors, or metastases occurring in these organs (8, 9) (**Figure 2**). It was also hypothesized that VCO is involved in the formation of clear cell renal cell carcinoma (RCC) (18). Future investigations will further uncover the relationship between VCO and clear cell RCC.

So far, approaches to study VCO are mainly focused on light microscopy and histopathological analysis of the growth pattern (15). Compared to angiogenesis, the molecular mechanisms of VCO are largely unknown and to-date, there are only a handful of papers available offering any mechanistic insights. That is the main reason why, thus far, no therapeutic strategies exist to inhibit VCO. Therefore, novel experimental strategies are warranted to gather detailed insights on this process, that can ultimately lay the foundation for development of therapeutics. Sequencing technologies, for instance, have revolutionized our understanding of cancer, and might be interesting to also study VCO-dependent tumors.

In this review, we highlight what is known to date about the models that can be used to study VCO, the underlying molecular mechanisms that drive VCO and their relevance in AAT resistance. Furthermore, we focus on the potential therapeutic strategies to target tumor VCO, based on newly gained insights into co-opted cell types. Lastly, we call attention to recently emerged approaches such as single-cell RNA sequencing and spatial transcriptomics and propose how these can be used in the future to further explore VCO (8, 17, 19, 20).

## Models demonstrating vessel co-option

### Evidence for vessel co-option in human cancers

During VCO, cancer cells migrate and hijack pre-existing blood vessels of the host organ in order to grow and to invade the surrounding tissue (**Figure 1**). This implies that cancer cells create their vascular supply in a non-angiogenic manner. Tumor VCO has first been described in lung cancer as an alveolar non-angiogenic growth pattern (3). Often, VCO in the lungs occurs in metastatic lesions (e.g. lung metastasis from renal cell carcinoma (15), but it can also arise in primary lung cancer (15, 21). In fact, co-optive or alveolar growth patterns have been observed in about half of the non-small cell lung cancers and in a third of small-cell lung cancers (22, 23). In addition to the occurrence in lung cancer, metastases in the lungs often grow *via* VCO. Here, metastases from colorectal and breast cancer have most often been associated with VCO, while lung metastases from RCC grow less frequently *via* VCO (9, 15). Notably, RCC has been known as one of the most angiogenic cancer types (24), thus it is very intriguing that the growth of RCC-derived metastases can partly rely on VCO, instead of angiogenesis.

Besides the discovery in lung cancer, co-option in tumors and metastasis has also been observed in many other organs including brain, liver, lymph node, colorectal and skin in patients (8, 25) (**Figure 2**). Gliomas are tumors in which VCO has been described the most (**Figure 2**). In human primary high-grade gliomas, cancer cells may surround brain capillaries or replace pericytes and astrocytes by adhering to the abluminal surface to co-opt pre-existing vessels which is referred to as ‘perivascular cuffing’ (25, 26). In primary low-grade gliomas, cancer cells co-opt vessels by infiltration of the parenchyma (27). In rat gliomas, it has been shown that tumors can grow by co-opting the pre-existing vasculature of the host. Upon co-option, the host vasculature does not use angiogenesis at first to support tumor growth but instead disintegrates, and thus creates an avascular tumor and impairs tumor growth. However, the remaining tumor recovers *via* robust angiogenesis by healthy vessels at the margins of the tumor (28).

In addition to primary brain tumors, VCO has also been identified in metastatic lesions in the brain, originating from distinct primary tumors. VCO was frequently observed in biopsy samples from patients with brain metastasis (from breast, melanoma, colorectal, and lung cancer) (29–31) (**Figure 2**). Moreover, assessment of brain metastasis from malignant melanoma revealed low vascular endothelial growth factor-A (VEGF-A) expression in the parenchyma, which led to an infiltrative phenotype, in which the pre-existing vasculature of the brain was co-opted. When the human melanoma cell line Mel57 was modulated to express recombinant VEGF-A-165, the metastases exhibited a fast infiltrative and expansion growth pattern with a marked central necrotic core and the co-opted peritumoral and intratumoral blood vessels were dilated causing an increase in vessel permeability (32).

In primary and metastatic liver tumors, cancer cells co-opt sinusoidal vessels and incorporate them in the tumor microenvironment, a process referred to as ‘replacement pattern’ (25, 33) (**Figure 2**). Here, the metastases grow without or with only minimal angiogenesis. In one study, for instance, liver specimens were resected from untreated tumors and examined by histology for their growth pattern. During replacement growth, tumors retain the basic architecture and cancer cells are well-differentiated and irregularly distributed, and the portal tracts are included in the tumor tissue (33). In addition to lesions from liver tumors themselves, metastases originating from different primary tumors are often found in the liver (**Figure 2**). Here, breast cancer liver metastases grow 90% *via* VCO, while there is less evidence of co-optive growth in colorectal carcinoma liver metastasis (16, 25, 34, 35). In lymph nodes, on the other hand, metastatic cancer cells derived from colorectal cancer, breast cancer, head or neck cancer can co-opt pre-existing vessels (25, 36–38) (**Figure 2**). Breast cancer metastases tend to grow in several organs *via* VCO, as for instance, VCO is also observed in more than 50% of metastatic cancer cells in breast cancer skin metastasis (39) (**Figure 2**). Lastly, melanoma cells have also been observed to co-opt blood vessels by cohesive migration on the abluminal surface in brain metastasis (32, 40–42) (**Figure 2**).

### Animal models to study vessel co-option

Multiple animal models have been described to study VCO including intracarotid, intracardiac, intravenous and orthotopic transplantation in mice, but also zebrafish and chick chorioallantoic membrane (CAM) models (**Table 1** summarizes the models demonstrating VCO per tumor type) (25). Importantly, the tumor vasculature is dependent on the anatomical site where the cell lines are injected (15). For instance, in several models, metastatic lesions from a particular tumor type use angiogenesis in one organ, but VCO in a different organ (15). Therefore, a limitation *in vitro* is that it is not possible to discriminate whether a cell line is classified for angiogenic or non-angiogenic tumor growth. In **Table 1**, we highlight known models, using different cell lines, that have been used to investigate the underlying mechanisms of tumor VCO.

**Table 1:** Summary of models demonstrating tumor vessel co-option per tumor type.

Cancer type	Species/ cell line	Model	Intracardiac/Intracarotid/Intravenous/Orthotopic transplantation	Zebra fish model	Chicken CAM & metastasis assay	Endothelial cell/tumor cell co-culture	Brain slice/ tumor cell co-culture	PMID
Breast	human	MDA-MB-231-LM	intravenous / orthotopic					Bridgeman et al. PMID: 27859259
	human	MDA-MB-231-BrM	intracardiac			X	X	Valiente et al. PMID: 24581498 Carbonell et al. PMID: 19516901 Voutouri et al. PMID: 30700544 Wang et al. PMID: 29130936
	human	MDA-MB-231 cells	intracardiac				X	Carbonell et al. PMID: 19516901
	murine (rat)	RBA adenocarcinoma						Holash et al. PMID: 10373119
	murine (rat)	MAT-B-III	intravenous					Szabo et al. PMID: 25319725
	murine	4T1 adenocarcinoma	intracardiac / intravenous / orthotopic	X	X		X	Bridgeman et al. PMID: 27859259 Carbonell et al. PMID: 19516901 Stoletov et al. PMID: 23321642
Colon	murine	C26	intravenous					Bridgeman et al. PMID: 27859259 Szabo et al. PMID: 25319725
	human	HT29 (colorectal)	orthotopic					Frentzas et al. PMID: 27748747
	human	HT25	intravenous					Szabo et al. PMID: 25319725
Fibrosarcoma	human	HT1080	intravenous					Szabo et al. PMID: 25319725
Glioma	murine (rat)	C6						Holash et al. PMID: 10373119
	murine	Cdkn2a−/−;hEGFRvII I					X	Griveau et al. PMID: 29681511
	murine (rat)	CNS-1						Voutouri et al. PMID: 30700544
	murine	GL26				X		Yadav et al. PMID: 27863376
	murine	GL261						Voutouri et al. PMID: 30700544
	murine	Olig2+ (Olig2cre/+;Trp53fl/ fl;hEGFRvIII)					X	Griveau et al. PMID: 29681511
	murine	Olig2− (Olig2cre/cre;Trp53f l/fl;hEGFRvIII)					X	Griveau et al. PMID: 29681511
	human	D54 GBM					X	Griveau et al. PMID: 29681511
	human	G55 GBM						Rubenstein et al. PMID: 11005565
	human	HF2303				X		Yadav et al. PMID: 27863376
	human	MGG8 GBM						Griveau et al. PMID: 29681511
	human	SF10417 oligodendroglioma						Griveau et al. PMID: 29681511
	human	U373 GBM					X	Caspani et al. PMID: 25032689
	human	U87 GBM					X	Caspani et al. PMID: 25032689
Liver	human	Hep3B-hCG	orthotopic					Kuczynski et al. PMID: 27059374
Lung	human	H2030-BrM adenocarcinoma	intracardiac			X	X	Valiente et al. PMID: 24581498 Er et al. PMID: 30038252
	murine	Lewis	intravenous					Holash et al. PMID: 10373119
Melanoma	murine	B16F10	intravenous		X	X		Szabo et al. PMID: 25319725 Stoletov et al. PMID: 23321642
	human	Mel57	intracarotid					Küsters et al. PMID: 11809675 Leenders et al. PMID: 15448011
	human	MDA-MB-435	intracarotid					Kienast et al. PMID: 20023634
	murine	D4M3A					X	Zhang et al. PMID: 31628560
	human	A7	intracardiac				X	Carbonell et al.PMID: 19516901
	human	A2058	intracarotid					Kienast et al. PMID: 20023634
Renal	murine	RENCA	intravenous					Bridgeman et al. PMID: 27859259

This table is modified from (25). All abbreviations can be found in the list of abbreviations.

Intracardiac injection of cancer cells occurs *via* the left ventricle of the heart in anesthetized mice and allows the cells to circulate in the body before reaching the microvasculature of the liver and lungs (20, 25, 43–45). This transplantation approach is mainly used to model brain and bone metastasis. Using this method, researchers discovered that plasminogen activator-inhibitory serpins can stimulate the survival of cancer cells (20, 25). In addition, by comparing transcriptomic signatures of brain metastatic subpopulation variants isolated from breast and lung cancer cell lines, they reported that L1CAM in brain metastases mediates VCO (20). In another study, the role of long noncoding RNAs in brain metastasis of breast cancer was studied after intracardiac injection of MDA-MB-231-Br cells and a specific long noncoding RNA (Lnc-BM) was documented to be essential for VCO in the brain (45).

Upon intracarotid injection, cancer cells are directly injected into the internal carotid artery of anesthetized mice to create experimental brain metastases. With this method, infiltrative co-optive growth patterns are visible after injection of human melanoma cell lines (M14, Mel57, 530) (32). VEGF-A regulates the progression of brain metastases of melanoma without inducing angiogenesis but by using the pre-existing vasculature (32). Melanoma brain metastases upon intracarotid transplantation also grew *via* VCO when mice were treated with AAT (46). Intracarotid injections of melanoma cells (A2058 and MDA-MB-435 human melanoma cells) led to perivascular growth using VCO in the resulting brain metastases, whereas injection of lung cancer cells with the same method caused angiogenic brain metastases, as assessed by multiphoton laser scanning microscopy (42).

Intravenous injection of cancer cells occurs *via* the jugular vein or tail vein and is mainly used to study lung metastasis. Jugular vein injection of mice with Lewis lung carcinoma cells causes lung tumors capable of co-opting the microvasculature (28). Also, experimental lung metastases generated after intravenous injection of several human and murine cancer cell lines (MAT-B-III rat mammary carcinoma, C26 murine colon carcinoma, HT25 human colon carcinoma, HT1080 human fibrosarcoma and B16 murine melanoma) showed that these metastases grow *via* VCO of the pulmonary vessels (47). Furthermore, when generating lung metastases models using murine renal adenocarcinoma cells (RENCA) and murine colon carcinoma cells (C26), VCO appeared to be a resistance mechanism to AAT (15). Tail vein injection of MDA-MB-231-LM cells (lung metastatic subpopulation) also causes cancer cells to spread perivascularly in the lung, mediated by neural cell adhesion molecule L1 (L1CAM) (44).

Orthotopic transplantation of cancer cells is performed by injecting them into specific anatomical sites and is commonly used to investigate the role of the microenvironment in tumorigenesis and metastasis (25, 48). Spontaneously developing metastases after orthotopic injection better recapitulate interactions between the tumor and the host as well as the specific characteristics of the whole metastatic process. In contrast to other methods, it takes more time to develop such metastases in mice. The perivascular growth of brain metastases was verified also in spontaneous metastases model of primary 4T1 mammary tumors (43). Moreover, MDA-MB-231-LM or MDA-MB-231^LM2-4^ cells co-opt alveolar capillaries when forming lung metastases after orthotopic mammary fat pad injections (15, 44). Injection of human HT29 colorectal cancer cells into a mouse liver generated advanced liver metastases growing *via* VCO. In this model, the importance of cancer cell motility during the co-opting process was documented (16). Moreover, orthotopic injection of Hep3B-hCG cells (human hepatocellular carcinoma) into mouse livers showed that VCO is used as an acquired resistance mechanism to AAT (49).

Zebrafish embryos are utilized to create metastases by injection of cancer cells into the cardinal vein (25, 50). Using these embryos helps to visualize early processes of brain colonization, like VCO and extravasation. Using this model, HMLE mammary cancer cells and 4T1 mammary cancer cells (murine) were shown to co-opt brain arteries, with a primary role of the connexin gap junction protein Cx43 in brain colonization and metastatic extravasation (50).

CAM *in vivo* models can also be used to explore how tumors grow and how they spread along blood vessels. A small hole is created on the top of the fertilized egg to allow the membrane to detach from the shell, followed by the placement of a matrigel containing a cancer cell mixture onto the CAM. After inoculation, spreading and growth of the cancer cells along the blood vessels is visualized by imaging. After a few incubation days, organs such as the liver or the brain can be isolated for imaging. In a study exploiting the chick embryo model, HMLE mammary carcinoma cells, B16 melanoma cells and 4T1 mammary cancer cells co-opt the blood vessels and several gap junction proteins (Cx43, Cx26) were found to play an important role during brain colonization and VCO (50).

Despite the limitations pointed out above, several *in vitro* models exist to study the cellular and molecular mechanisms of VCO by cancer cells. For instance, EC - cancer cell co-cultures have been used to study the interaction between blood vessels and cancer cells (25, 40, 45). ECs can be stimulated to create capillary-like structures (but these are not perfused like *in vivo* blood vessels) and can be monitored how cancer cells migrate along the vessels by time-lapse imaging (25). Another model that can be used *in vitro* is brain slice - cancer cell co-culture (11, 20, 25, 26, 43, 45, 51). This model, which has been used to study molecular and cellular processes in the microenvironment of the brain, is generated by cutting brain slices with a vibratome. Brain slices are then cultured onto porous membrane inserts in the brain slice medium and incubated for a short time. Afterwards, cancer cells are pipetted onto the brain slice surface and the interaction between brain blood vessels and cancer cells can be visualized by using fluorescence microscopy (25).

This method was used in a study, in which the authors plated human and murine cancer cells (A7, K1735M2, MDA231BR, MDA-MB-231, 4T1 cells) onto murine brain slices and observed that cancer cells spread rapidly along the blood vasculature (43). Human U87 and U373 glioblastoma multiforme (GBM) cells that were seeded onto brain slices were also shown to co-opt the pre-existing vasculature (26). When culturing human D54 GBM cells onto brain slices, bradykinin appeared to be critical for the perivascular migration of brain cancer cells (11). Moreover, when low and highly invasive breast cancer cells (MDA231 vs. MDA231-BrM) and lung adenocarcinoma cells (H2030 vs. H2030-BrM) were seeded onto brain slices, low-invasive brain parental cancer cells succumbed *via* serpin- and plasmin-dependent pathways, whereas brain invasive BrM cells moved towards and disseminated over brain capillaries (20). Brain slices were also used to investigate the roles of Wnt7 in glioma cells during VCO. The authors discovered that Olig2^+^ (Wnt7 wildtype) glioma cells used VCO, while Wnt7 null glioma cells did not migrate along blood vessels *via* VCO (52). Furthermore, co-option was arrested when Wnt signaling was inhibited in SF10417 human oligodendroglioma cells and Cdkn2a^-/-^;hEGFRvIII glioma progenitor cells. In addition, murine D4M3A melanoma cells (established from the metastatic melanoma Tyr::CreER;BrafCA;Ptenlox/lox mouse model (53)) plated onto brain slices from immunocompetent VE-Cadherin reporter mice (Cdh5-CreERT2;ZSGreenloxp/stop/loxp) mice, only migrated along the pre-existing vasculature of the brain (25, 52). Overall, while extremely helpful in studying VCO mechanisms, one should consider that the microenvironment and culture conditions from *in vitro* models are different from the *in vivo* microenvironment, therefore the mentioned results have to be verified with *in vivo* models.

Together, these *in vivo* and *in vitro* models have proven their value in unraveling possible VCO molecular mechanisms and their response to therapies. However, there are still limitations associated when using these models. To give some examples, for the intracardiac transplantation for instance, the efficiency of metastasis generated is low and dependent on the cell line used (25). A limitation of the intracarotid approach is that the carotid artery is ligated, potentially causing injury which might influence VCO (25). For orthotopic transplantation, a limitation is that it takes a long time to create metastases compared to the other methods described (25).

## Molecular mechanisms of vessel co-option and vessel co-option as resistance mechanism to anti-angiogenic therapy

### Cancer cell – EC interactions

**Figure 3:**
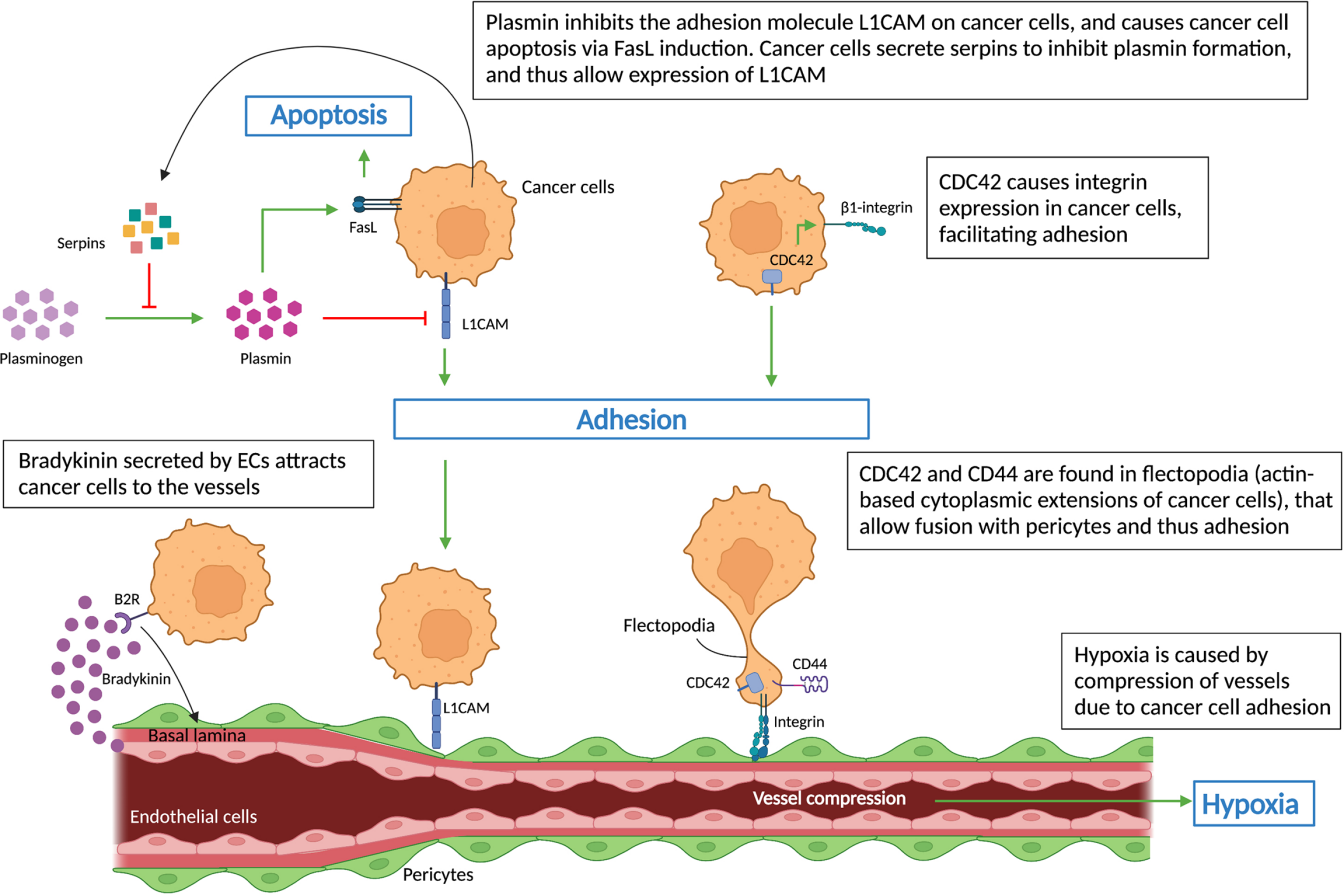
Cell-cell interactions during vessel co-option. Schematic graph showing key regulators (L1CAM, CDC42, CD44, integrins, serpins) of non-angiogenic, co-opted cancer cells, which cause cancer cells’ adhesion to vessels – a hallmark of vessel co-option. Cancer cells’ attachment to the pre-existing vessels leads to vessel compression, which in turn results in hypoxia. All abbreviations can be found in the list of abbreviations.

As defined, VCO relies on an interaction between cancer cells and the host vasculature, directly with ECs or indirectly *via* pericytes. Therefore, understanding these cell-cell interactions is key to understand cancer cell invasion *via* VCO (**Figure 3**). The phenotypic features of non-angiogenic cancer cells include reduced adhesion to each other and to the extracellular matrix (ECM), together with increased motility and invasion (8, 11, 12). To date, only a few proteins and mechanisms are proposed for their involvement in the formation and progression of tumors relying on VCO.

Bradykinin, the ligand of the B2 receptor (B2R), a G-protein coupled receptor, has been strongly associated with directing invading glioma cells toward blood vessels (11). B2R is highly expressed on glioma cells isolated from patient biopsies and causes their migration towards pre-existing blood vessels *via* a bradykinin gradient released by blood vessels (**Figure 3**), as demonstrated in rat brain slices (54). Inhibition of bradykinin in turn reduced the numbers of cancer cells associated with blood vessels (54).

Tumors and metastases relying on VCO are often characterized by a large hypoxic core (54), suggesting that hypoxia-driven mechanisms may impact VCO. For instance, hypoxia enhances prolyl-4-hydroxylase α1 (P4HA1) expression, which can convert bradykinin to hydroxyprolyl-bradykinin, as shown in human pancreatic cancer (55). In this study, circulating hydroxyprolyl-bradykinin/bradykinin ratios measured in patient plasma samples mirrored local tissue hypoxia regulated by hypoxia-inducible factor 1 (HIF1α). In fact, while further studies are required, these results may propose circulating hydroxyprolyl-bradykinin/bradykinin ratios as indicators of tumor hypoxia, patient prognosis and treatment responses (55).

Highly invasive, motile cancer cells are another feature of co-opted tumors and metastases. Cancer cell migration/invasion can be affected by components of the plasminogen-plasmin system (56). In an animal model of brain metastasis, L1CAM was identified in metastatic cells, where it assists their adherence to brain capillaries. The study demonstrated that cancer cells also expressed serpins, including neuroserpin and serpin B2, which inhibits stromal cell-derived plasmin – an L1CAM inactivator, and inducer of cancer cell death *via* FasL signaling (**Figure 3**). Thus inhibition of plasmin by serpins promotes their adhesion to brain vessels *via* L1CAM, reduces tumor apoptosis and induces VCO and metastasis (20) (**Figure 3**).

Cancer cells, after moving along pre-existing vessels, adhere to the basement membrane of ECs or pericytes in order to exploit VCO (57). Therefore, the interaction between cancer cells and vascular cells is a determining factor for the formation of co-opted lesions. Integrins are ECM adhesion molecules that mediate not only cell-cell interactions but also cell-ECM interactions, which are involved in metastasis and drug resistance (58, 59). β1 integrin levels on metastatic brain cells regulate their interaction with different components of the basal lamina from brain capillaries such as fibronectin, laminin, vitronectin, and collagen I and IV (60). In breast cancer, β4 integrin plays a similar role in the cancer cell – ECM interaction (43, 60, 61). Moreover, β1 integrin is also the main target of cell division control protein 42 homolog (CDC42), a member of the Rho GTPase family associated with actin-dependent cytoplasm extension regulation (**Figure 3**), which was shown to mediate cancer cell - EC interactions (62). Interestingly, novel treatments using small molecules and miRNAs to inhibit the abnormal expression of CDC42 may slow down the metastasis process (62, 63). In addition, CDC42, together with cell adhesion molecule CD44, were found to be crucial for the motility of cancer cells as they are highly concentrated in the flectopodia, the actin-based cytoplasmic extensions on the cancer cell membrane that impact the contractile activity of pericytes (**Figure 3**). Cancer cells fuse with pericytes *via* flectopodia creating a hybrid cell that expands the tumor margin, thus promoting VCO and tumor-related hypoxia by vessel constriction (26, 64) (**Figure 3**).

### Signaling pathways leading to vessel co-option

**Figure 4:**
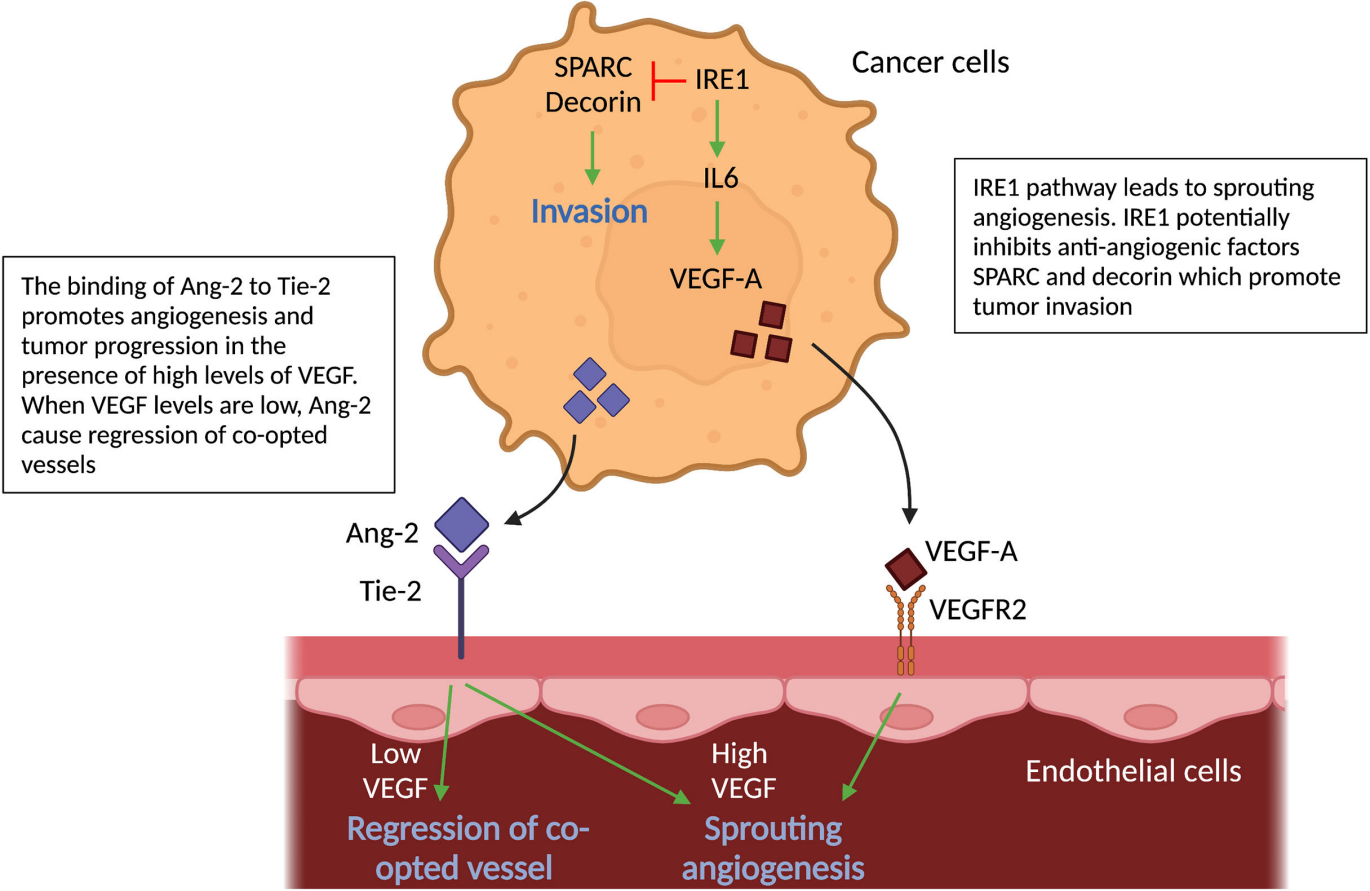
Molecular pathways in cancer cells and vascular cells driving angiogenesis. Schematic graph showing the known pathways associated with angiogenesis, the inhibition of which has been associated with vessel co-option. *Ang2-Tie signaling:* Signaling of Ang-2 through its receptor Tie-2 can cause sprouting angiogenesis if VEGF levels in the tumor microenvironment are high. If VEGF levels are low, Ang-2- Tie-2 signaling leads to regression of co-opted vessels. IRE1 signaling: IRE1 in cancer cells promote VEGF production, which can induce sprouting angiogenesis. On the other hand, it is indicated, that IRE1 impedes cancer cell invasion *via* inhibition of anti-angiogenic factors, such as SPARC and Decorin. All abbreviations can be found in the list of abbreviations.

A theoretical model postulates that, to establish VCO, cancer cells first invade the tissue parenchyma, displace existing healthy tissue cells, and then interact with the vascular cells (65). These cancer cell-EC interactions during VCO trigger molecular alterations in both cell types that are crucial for the motility, adhesion and invasion of cancer cells to pre-existing vessels (**Figure 4**). Here, we highlight signaling pathways in co-opted cancer cells and co-opting ECs that have been associated with VCO.

The vascular growth factor angiopoietin-2 (Ang-2), which can be secreted by cancer cells (amongst others), is known to cause regression of co-opted blood vessels (58). Ang-2 is a ligand of the endothelial tyrosine kinase-receptor (Tie-2) (66). Binding of Ang-2 to its Tie-2 receptor results in a loss of vascular integrity and an increase in vascular permeability (66). Furthermore, in the presence of high levels of VEGF, Ang-2 leads to EC proliferation and triggers sprouting angiogenesis; in contrast, when VEGF levels are low, Ang-2 causes loss of vascular structures with marked regression of co-opted vessels. Ang-2 therefore may inhibit VCO in tumors (67–70) (**Figure 4**).

Moreover, inositol-requiring enzyme 1 (IRE1), an endoplasmic reticulum transmembrane stress sensor and a pivotal mediator of the unfolded protein response, plays a role in VCO (71). Using the CAM model and a murine orthotopic brain transplant model, reduced expression of IRE1 in glioma cells downregulates pro-angiogenic factors (VEGF-A; Interleukin-6 (IL-6)) and upregulates anti-angiogenic factors (such as SPARC and Decorin) linked to mesenchymal differentiation, tumor invasion and VCO (71). Thus, one may suppose IRE1 inhibits these anti-angiogenic factors (**Figure 4**). In addition, ectopic expression of IL-6 in IRE1-deficient tumors restores the angiogenic phenotype and neutralizes VCO, however it does not reverse cancer cell infiltration (71) (**Figure 4**).

**Figure 5:**
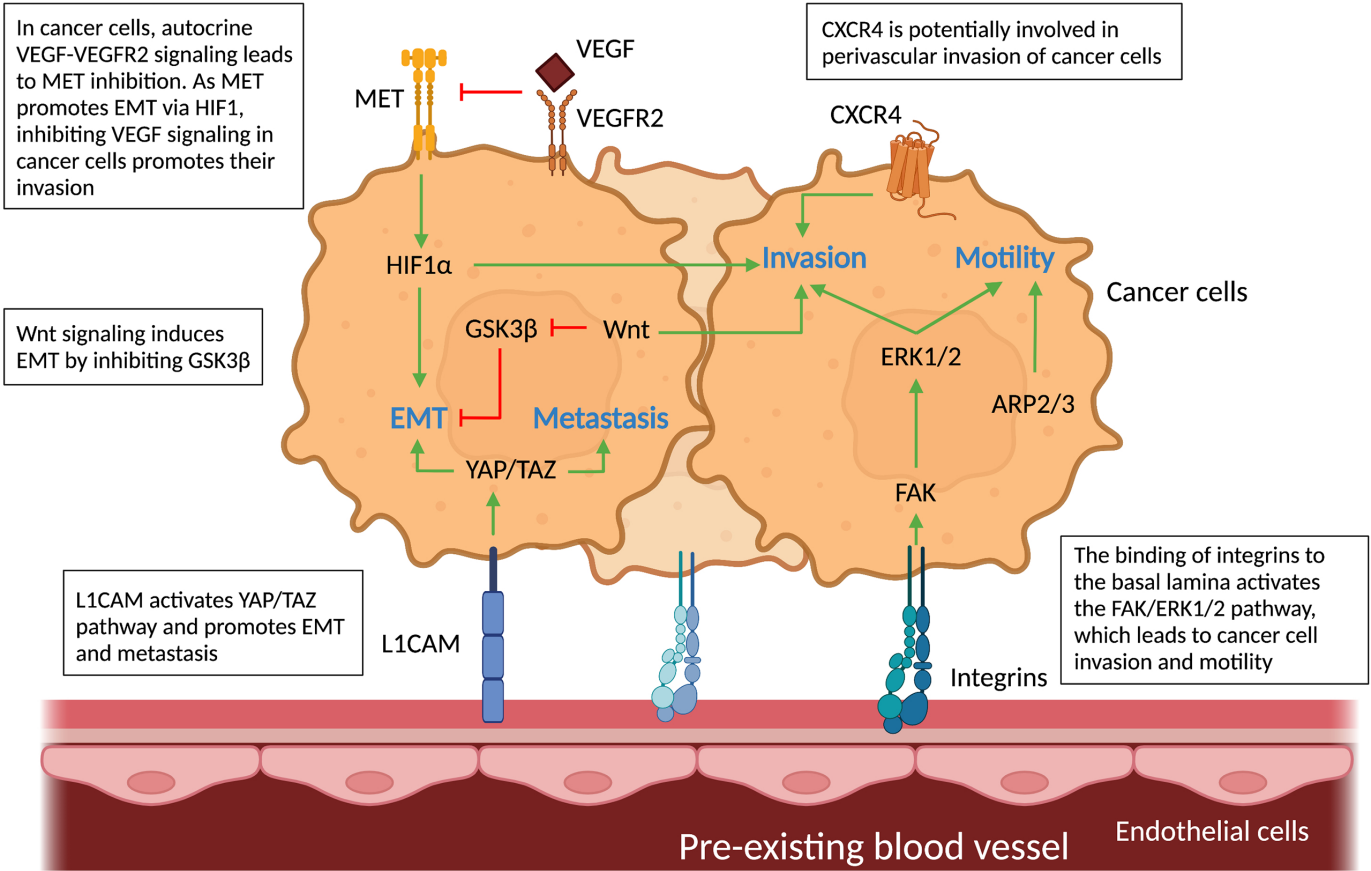
Molecular pathways in cancer cells and vascular cells driving vessel co-option. Schematic graph showing the known pathways associated with vessel co-option (VCO) and how they relate to each other creating a complex network. *Top: Cancer cells:* The pathways involved in VCO in cancer cells and their roles are shown: metastasis (YAP/TAZ), invasion (HIF1α, FAK-ERK1/2, Wnt, CXCR4), motility (FAK-ERK1/2, ARP2/3) and EMT (GSK3β, MET, YAP/TAZ). *Bottom: Tumor vessels:* Binding of cancer cells to tumor vessels *via* integrins and L1CAM can result in motile and invasive cancer cell phenotypes. All abbreviations can be found in the list of abbreviations.

Several signaling pathways may underlie the migratory, invasive characteristics of co-opted cancer cells. As invasive cancer cells are regularly associated with an epithelial-to-mesenchymal transition (EMT)-like phenotype, it comes to no surprise that some of the proposed mechanisms are related to EMT in cancer cells. During EMT, cancer cells lose their epithelial features and acquire mesenchymal features, thereby becoming less adherent to each other and more migratory (72). In the following, we list downstream signaling in cancer cells related to a migratory EMT-like phenotype and VCO (**Figure 5**).

First, as explained in the previous section, integrins play a key role in cell-cell interaction, but they also activate downstream pathways related to VCO. For instance, the interaction between cancer cells and ECs through β1 integrin leads to the activation of focal adhesion kinase (FAK) and extracellular signal-regulated kinase (ERK) 1/2 in co-opting cancer cells. FAK, a tyrosine kinase highly expressed in GBM cells, has been associated with cell motility and cancer cell invasion – features of tumor VCO (43, 73) (**Figure 5**). Whether the induction of FAK *via* cancer cell-EC interaction indeed leads to VCO remains to be further validated. Second, CXC-chemokine receptor 4 (CXCR4) signaling is potentially critical during VCO (**Figure 5**), as this pathway is involved in perivascular invasion of brain-metastasizing cancer cells through stimulation by brain EC-derived CXC-chemokine ligand 12 (CXCL12) (25, 74). In fact, CXCR4 was found highly expressed on metastatic glioblastoma cells relying on VCO (74).

A third possible signaling pathway is the Yes-associated protein (YAP) – transcriptional co-activator with PDZ-binding motif (TAZ) pathway (75–77). YAP and TAZ are primary sensors that play a crucial role in various aspects of cancer progression including promoting an EMT-like cancer cell phenotype and metastasis (75–77). Interestingly, L1CAM, known for its roles in VCO, activates the mechanotransduction effector YAP (44). It is moreover known that L1CAM-mediated pericyte-like spreading plays a major role in the initiation of metastasis (44). Further studies are required to directly link YAP-TAZ signaling in cancer cells to L1CAM1-dependent VCO. Fourth, the characteristic hypoxia of co-opted tumors generates high levels of HIF1α, a known inducer of the EMT transcription factor Zinc finger E-box binding homeobox 2 (ZEB2) that suppresses ephrinB2, thereby enhancing tumor invasiveness (78) (**Figure 5**). Lastly, canonical Wnt signaling in non-angiogenic cancer cells can induce an EMT-like phenotype by inhibition of the glycogen synthase kinase 3β (GSK3β), which leads to increased Snail stability (8, 79–81). Importantly, a subtype of Olig2^+^ oligodendrocyte-like glioma cells was shown to upregulate Wnt7 expression and promote invasion of cancer cells *via* co-option of existing brain vessels, linking Wnt7 signaling to VCO (52) (**Figure 5**).

### Vessel co-option as resistance mechanism to anti-angiogenic therapy

A link between AAT resistance and VCO has been demonstrated in different types of tumors such as brain, colorectal, breast, renal and liver cancer (9, 15, 46, 49, 82, 83). For example, treatment with Bevacizumab (an anti-angiogenic drug that targets VEGF-A), used to treat kidney, colon, rectum, lung, or breast tumors, and some brain tumors, causes a metabolic shift toward glycolysis making the remaining glioblastoma cell populations less dependent on angiogenesis and promoting the VCO typical pro-invasive phenotype in a subset of GBM patients (84, 85). Thus, drivers of these invasive (EMT-like) cancer cell phenotypes upon AAT are likely associated with VCO. For instance, in an *in vivo* liver metastasis model, AAT induces the actin-related protein 2/3 complex (ARP2/3), which promotes cancer cells motility and VCO (16) (**Figure 5**). Another pathway that might be involved in VCO is the hepatocyte growth factor (HGF) signaling mediated through binding to the tyrosine kinase receptor MET. VEGF inhibition is accompanied by activation of MET, which is correlated with poor prognosis and resistance to therapy (82, 86–92). An EMT phenotype could be induced in GBM cells upon genetic or pharmacologic VEGF inhibition in an HGF-MET dependent manner (85) (**Figure 5**). Importantly, invasion and metastasis were induced by inhibiting VEGF signaling pathways, which could be impeded by concurrent MET inhibition (85, 89). Furthermore, VEGF inhibition, in addition to increasing MET levels, leads to enrichment of hypoxia-associated markers (including HIF1α (**Figure 5**), carbonic anhydrase 9 (CA9) and GLUT1), as well as downregulation of epithelial adherens junction proteins T-cadherin and E-cadherin, thus creating more mesenchymal, invasive cancer cells (85).

More functional studies are required to identify the underlying drivers of VCO. It is important to note that many of the described signaling pathways are only loosely linked, or even only hypothesized to be involved in the VCO process, and experimental evidence providing direct links to tumor VCO is yet to be imparted. Notably, the roles of EMT in VCO are not yet fully understood, and the existing links need further strengthening (65, 72). Moreover, while VEGF inhibition has been often connected to tumor VCO, VEGF is also known to downregulate cancer cell invasion *via* enhanced recruitment of the protein tyrosine phosphatase 1B (PTP1B, which can directly dephosphorylate MET and VEGFR2), thereby suppressing cancer cell migration (85). Nonetheless, these initial insights are a promising start to better characterize the cellular and molecular mechanisms of VCO.

## Potential therapeutic anti-tumor vessel co-option strategies

**Figure 6:**
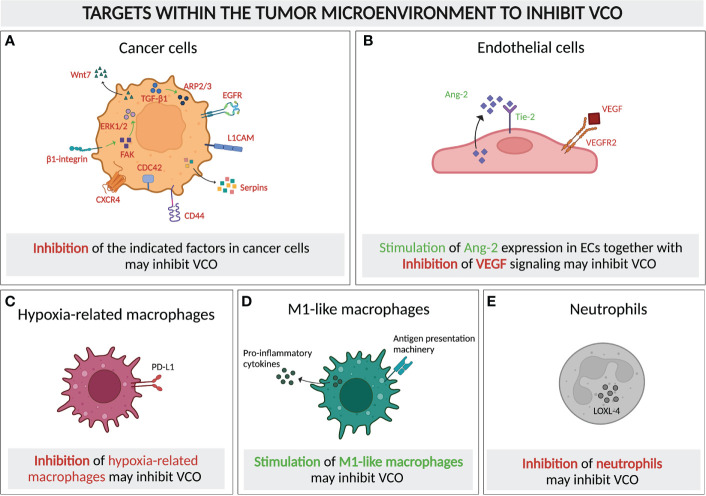
Molecular and cellular targeting to inhibit vessel co-option. Schematic graph showing the cellular components of a typical VCO-related tumor microenvironment and their potential as targets to inhibit VCO, either by inhibiting **(A–C)** or promoting the presence and/or signaling of these cells. *Targets for inhibition:* **(A)** Tumor cells with signaling pathways and molecules for potential targeting highlighted (TGF-β1, ARP2/3, EGFR, L1CAM, Serpins, CD44, CDC42, CXCR4, β1-integrin, FAK, ERK1/2, Wnt7), **(C)** Hypoxia-related macrophages (PD-L1 as potential target) and **(E)** Neutrophils (LOXL-4 presenting a potential target) *Targets for dual inhibition and stimulation:* **(B)** ECs could potentially dually targeted: VEGF signaling could be inhibited, while simultaneously promoting Ang-2 signaling. = *Targets for stimulation:* **(D)** M1-like macrophages should be promoted. All abbreviations can be found in the list of abbreviations. *All figures were generated with Biorender.com*.

As mentioned above, there is accumulating evidence linking VCO to resistance to AAT. In fact, one may also speculate whether the failure of several phase III clinical trials for various anti-angiogenic drugs (93) could be (partly) caused by tumors switching to VCO as alternative mode of vascularization. Here, cancer cells may potentially reside along existing vessels for survival and switch into a state of temporary dormancy. While these intriguing questions have yet to be investigated, targeting of VCO may add a significant contribution in developing novel anti-cancer therapies, in particular when combined with AAT. To develop potential strategies for VCO inhibition, it is important to understand which cell types in the tumor microenvironment can be targeted, as well as how we could target those cells (**Figure 6**). Here, we are proposing some potential targets and approaches for VCO inhibition, many of which are based on the mechanisms suggested in the previous section.

### Targeting cancer cells

As the majority of studies on VCO has centered around molecular mechanisms of co-opted cancer cells, targeting those cells to achieve VCO inhibition has attracted attention, in particular focusing on motility or adhesion pathways. The invasion of cancer cells to the perivascular environment relies on their motility, in which motility-involving molecules such as ARP2/3, L1CAM, serpins, CD44, CDC42, CXCR4, epidermal growth factor receptor (EGFR), as well as Wnt7 signaling, are critical players (16, 20, 25, 26, 43, 52, 64, 73, 74, 94–96) (**Figure 6A**). Knockout of a subunit of ARP2/3 suppressed VCO in a preclinical model of advanced colorectal cancer lung metastasis (CRCLM) (16). The inhibition of VCO was demonstrated by a decrease in cancer cell migration and a replacement histopathological growth pattern (HGP), where VCO is exploited to ensure the blood supply (16, 34, 97). Moreover, the upregulation of ARP2/3 in VCO-dependent cancer cells relies on runt-related transcription factor-1 (RUNX1), which is expressed upon the induction of transforming growth factor beta-1 (TGF-β1) (98, 99) (**Figure 6A**). In addition, L1CAM and serpins, the adhesion-assisting molecules expressed by metastatic cells, are other potential targets due to their cruciality in the formation of vessel co-opted metastases (20) (**Figure 6A**). Another interesting approach could be to suppress CD44 and CDC42, which might impair VCO by impeding the cancer cell-pericyte fusion (**Figure 6A**). This double inhibition has also been shown to facilitate pericyte conversion into a phagocytic/macrophage-like phenotype, favoring an innate immune response against the tumor (26, 64). Intriguingly, targeting CXCR4, a crucial molecule for brain microvasculature invasion by glioblastoma cells, suppresses tumor invasion and renders tumors sensitive to radiation therapy (25, 74), thus presenting another potential intriguing target (**Figure 6A**).

As few studies investigated the roles of different molecules in VCO upon AAT, combinatorial treatment approaches, integrating AAT with blockade of VCO can be considered. For instance, AAT *via* VEGFR2 blockade reduced intracerebral glioblastoma growth, but caused an increase in tumor migration with a VCO pattern. Interestingly, this increase in cancer cell migration could be inhibited by combined treatment with VEGFR2 and EGFR antibodies, as was demonstrated by a decreased migration of glioblastoma cells in an *in vitro* system (94, 95). Furthermore, Wnt7, which is critical for VCO in Olig2^+^ glioma cells, showed an increased expression in glioma preclinical models and patients after anti-VEGF treatment. Use of Wnt7 inhibitors was efficient to prevent vessel contact of glioma cells, thus limiting the metastasis of vessel co-opted cancer cells (52) (**Figure 6A**). The β1-integrin subunit mediates the adhesion of metastatic cancer cells to the vascular basement membrane of brain blood vessels. Blockade of this subunit in cancer cells could prevent their adhesion to the membrane of vascular cells, thus attenuating the establishment and growth of metastasis (43) (**Figure 6A**). Moreover, inhibition of ERK1/2 and FAK, the downstream molecules of β1-integrin, were associated with a beneficial outcome when combined with AAT (43, 73, 96, 100–102) (**Figure 6A**).

### Targeting the tumor microenvironment

Tumors not only contain proliferating cancer cells, but comprise other cell types such as vascular cells, immune cells and a variety of connective-tissue cells, which make up the tumor microenvironment (103, 104). To date, molecular targeting of cancer cells to overcome VCO by preventing their mobility and adhesion towards the vasculature has caught considerable attention. However, strategies targeting other non-tumoral cells could be further exploited, in particular with the outlook on single-cell RNA studies on VCO in the future (as outlined in the next section), which may yield additional insights into the vessel co-opted tumor microenvironment.

Once tumors commit to VCO, an effective way of preventing tumor progression would be to target the existing tumor vasculature. In vessel co-opted tumors, Ang-2 is overexpressed by mature ECs (67). Ang-2, together with VEGF, promotes angiogenesis and tumor progression (67, 105). However, in the absence of VEGF, overexpression of Ang-2 leads to EC apoptosis, vessel destabilization and vessel loss, thus generating a hypoxic core which induces cancer cell apoptosis (106, 107). Therefore, the combination of anti-VEGF together with enhancing the endothelial Ang-2 expression might inhibit the progression of cancer cells along the existing vasculature (**Figures 4**, **6B**).

While additional targeting of the tumor vasculature could be considered a potential approach to overcome VCO (upon AAT), a recent single-cell study investigating VCO in preclinical models discovered that both co-opted ECs and pericytes display gene expression signatures highly similar to those in their healthy counterparts (10). This raises the question whether targeting co-opted ECs and pericytes may be a desirable option, considering the possible effects on the healthy vasculature (10). On the other hand, the observed similarities in transcriptomic profiles of vascular cells of healthy and co-opting vessels may only apply to the investigated models, and further studies may unravel vascular differences of co-opted vessels compared to their healthy counterparts, when exploring different murine models, or in the clinical context. Interestingly, using a nanoparticle contrast agent in Computed Tomography Imaging in a murine model of breast cancer, co-opted vasculature was identified exhibit high vessel wall permeability (108). Thus, a leaky vasculature as a distinguishing characteristic of VCO-dependent tumor could hold great potential for future targeting strategies.

Immune cells in the tumor microenvironment might present another target to inhibit VCO. Tumor-associated macrophages, with both anti- and pro-tumor subsets, play pivotal roles in either promoting or inhibiting tumor surveillance and therefore tumor development (10, 109). An enrichment of immunosuppressive hypoxia-regulated macrophages was found in a preclinical VCO model, which likely resulted from the increased hypoxia level in tumors relying on VCO (10). Thus, inhibiting these cells may help regulate VCO (**Figure 6C**). Interestingly, M1-like macrophages, which present tumor-associated antigens and express pro-inflammatory cytokines, and thereby activate cytotoxic T lymphocytes, were highly detected in the same VCO-dependent tumor (10) (**Figure 1**). The enrichment of these pro-inflammatory macrophages may indicate that VCO can actually lead to anti-tumor immunity, which, however, was not strong enough to shrink the tumors, probably due to the existence of other immunosuppressive hypoxia-regulated and M2-like subsets. This raises the question whether further promoting M1-like macrophage polarization could potentially help to improve the regression of vessel co-opted tumors (**Figure 6D**). Indeed, a mathematical model of glioma suggested that enhanced oxygenation level could promote M1 abundance in tumors (110), while limiting the accumulation of the immunosuppressive hypoxia-related subset of macrophages. Similar to tumor-associated macrophages, tumor-associated neutrophils are also known for both their pro- and anti-tumor growth promoting roles (111). The accumulation of lysyl oxidase like 4 (LOXL-4)-expressing neutrophils in colorectal cancer lung metastases with replacement growth pattern, where VCO is the dominant pattern of vascularization, suggested their possible role in supporting the growth of vessel co-opted tumors, and indicated them as potential targets to control VCO (112) (**Figure 1**, **Figure 6E**). These studies suggest that targeting immune compartments of the tumor microenvironment might potentially contribute to VCO inhibition, thereby overcoming AAT resistance. The challenge will obviously be to selectively target VCO-promoting immune cells, while not eliminating anti-cancer immune cells.

Interestingly, cells in the brain stroma such as astrocytes and microglia express plasmin to suppress the adhesion-promoting activity of L1CAM produced by metastatic cancer cells (20). As explained in the above sections, plasmin is however suppressed by tumor cell-secreted serpins, such as neuroserpin and serpin B2. Thus, targeting serpins or enhancing plasmin production in the tumor microenvironment awaits further exploration to limit the adherence of cancer cells to the existing vessels, thereby preventing the formation of vessel co-opted tumors.

Healthy cells adjacent to the lesions also play a role in the tumor displacement process. When cancer cells approach the existing vasculature, their growth relies on the invasion of vessel-adjacent healthy tissue. Therefore, cells in close proximity to VCO lesions must undergo phenotypic alterations so that the displacement by cancer cells could occur. In a VCO-dependent CRCLM model, apoptosis, motility and EMT were induced in tumor-adjacent hepatocytes by the cancer cells *via* upregulation of proapoptotic cleaved caspase-3 and cleaved PARP-1, EMT-related vimentin and motility-mediated ARP2/3 (113). This resulted in hepatocyte displacement and formation of vessel co-opted metastases. Thus, the interactions between tumor and healthy cells should be further explored to identify potential strategies to interrupt this tumor promoting liaison.

### Potential therapeutic approaches

As mentioned above, different cell types are involved in VCO-related tumorigenesis and tumor progression, thus it is essential to design an approach that specifically targets involved cells but shows no specificity to other cell types, to avoid/minimize off-target effects. Gene therapy using tumor-specific promoters has shown potential to suppress or to enhance the expression of genes in cancer cells with promising delivery and expression efficacies (114). Potential tumor-specific promoters such as human telomerase reverse transcriptase, α-fetoprotein promoter, thyroid transcription factor 1, or Mucin 1, are activated by a malignant process, but not in normal healthy cells (114–116). Gene delivery mediated by viral vectors has been exploited in clinical trials (117, 118). Recently, gene delivery systems using non-viral vectors have been developed for cancer therapy, in which gene carriers including lipids, polymers or peptides are investigated to transfer nucleic acids into target cells with low toxicity (119). By using non-viral gene carriers, limitations in delivery capacity and immunogenicity of classic engineered viruses can be overcome, as various types of nucleic acids could be packed in the novel carrier system (119, 120). The benefit of this system is the flexibility in carriers’ structure, which could be modified to increase their specificity and targeting (121). Thus, while partly still in development, opportunities exist that enable modification of molecular targets in individual cell types. Therefore, one may consider such strategies to seek blockade of motility- and adhesion-related molecules in cancer cells, or inducing a “hot” immune environment (e.g. containing immune cell populations with anti-tumor activities) in vessel co-opted tumors (4, 110, 122).

As tumors can switch between VCO- and angiogenesis-driven oxygenation (123), the combination of AAT with VCO inhibition may present a viably strategic approach to overcome AAT resistance and to improve cancer therapies. Interestingly, mathematical modeling of VCO indicated that sequential treatment of VCO inhibition followed by VEGFR blockade could reduce the tumor burden compared to simultaneous treatment (110). Notably, in this theoretical approach, drugs interfering with VCO were modeled computationally. It was also calculated that blocking both VEGF and VCO could enhance tumor oxygenation and increase M1 macrophage abundance, thus improving therapeutic outcomes (110). However, due to the heterogeneity between different tumors, more insight in the relationship between angiogenic and VCO tumor growth is needed to design specific treatments for each cancer type.

The immunosuppressive molecules programmed-cell death ligand 1 (PD-L1)/PD-L2 are upregulated in various cell types in the tumor microenvironment, including cancer cells, macrophages, lymphocytes and ECs among others (124–126). Moreover, in VCO-dependent tumors, PD-L1 and PD-L2 were respectively enriched in hypoxia-related and antigen-presenting/inflammatory macrophages (10). This observation underscores that targeting of non-vascular cell types in VCO tumors may present an attractive approach. Therefore, combining AAT with immunotherapies could result in a better antitumor effect, as the tumors’ nutrient and oxygen supply as well as their immune evasion mechanism are inhibited simultaneously. Indeed, there is increasing evidence of successful combination therapies of AAT and anti-PD-1/PD-L1 blockade when they were used in preclinical studies, partly proving their potential (127–130). Recently, this strategy was validated in several phase III clinical trials, where anti-angiogenic drugs combined with immune checkpoint inhibitors resulted in significant response rate and increased overall survival in cancer patients (131, 132).

It is important to note that such trials were originally designed on the premise that (i) VEGF signaling in angiogenic tumors can induce immunosuppression (133), and (ii) AAT causes vessel normalization, thereby enabling better intra-tumoral drug delivery. To what extent the success of these recent trials involves inhibition of VCO remains to be determined. Another interesting aspect to consider may be the emerging role of ECs as active participants in tumor immunity (134). For instance, several EC subsets, such as liver sinusoidal endothelial cells (135), or a fraction of ECs in lung cancer (136) were shown to express PD-L1, thereby exhibiting the potential to directly modulate immune cells. It will thus be intriguing to unravel how such types of ECs are impacted by immune checkpoint blockade, and what impact this targeting might have with respect to VCO-dependent tumors.

For most tumor types, chemotherapy has been considered the first-line of treatment for decades. Interestingly, some studies demonstrated a possibility to reduce vessel co-opted tumor growth when combining chemotherapy with AAT. Local invasion of metastatic breast tumors, which was worsened by AAT treatment alone, was blocked upon concurrent combination of AAT and Paclitaxel (137). The chemotherapy drug Topotecan improved the efficacy of AAT in a VCO-dependent triple negative breast cancer model (138). Therefore, combination of both therapies could be another option to overcome VCO and AAT resistance.

As it becomes obvious, while several possible (combinatorial) treatments might be considered and further investigated, an in-depth knowledge about all co-opted cell types and the mechanisms driving VCO is required to efficiently revise novel treatment strategies.

## Approaches to study vessel co-option: old *versus* new

To date, approaches to study VCO have mainly been reduced to the identification of histological characteristics by light microscopy. The utilization of only one major methodology to investigate VCO, in addition to the scarcity of proposed molecular mechanisms, call for the exploitation of alternative novel experimental techniques, to shed more light on this thus far mysterious process.

### Bulk and single-cell RNA sequencing technologies

Sequencing technologies have revolutionized our understanding of cancer and other diseases, by enabling insight into cells’ genomes, methylomes and transcriptomes in different contexts, leading to the discovery of novel molecular disease mechanisms, and vulnerabilities for potential targeting. However, sequencing studies focusing on VCO are rare. A few studies including RNA arrays, or bulk sequencing on cancer cells treated with AAT may hold some indications for changes in the transcriptome of co-opted cancer cells. In a 2009 study, glioma cells treated with AAT were subjected to a quantitative real-time reverse transcription PCR array (19), revealing a few transcriptional changes in cancer cells in co-opted tumors. The most upregulated genes in these glioma cells were metalloproteinase (MMP)-12, MMP-9, collagen type IV, α3 (COL4-A3), and CXC-chemokine ligand 9 (CXCL9), while laminin α1 (LAMA1), integrin β2 (ITGB2), MMP-1, and hyaluronan synthase 1 (HAS1) were downregulated after Bevacizumab treatment. Thus, in these cancer cells, genes related to angiogenesis seemed highly upregulated. Moreover, the deregulation of genes related to ECM remodeling may contribute to the increased invasive properties of the cancer cells. Importantly, when using the glioma cells in an *in vivo* model, alternative angiogenic mechanisms were found as resistance mechanisms, and VCO was not investigated, although increased invasiveness of the cancer cells was noted.

Recently, using animal models of VCO in lung metastasis, single-cell transcriptomic technology was used for the first time to identify the transcriptome of co-opted cell types (111). In both models of AAT-induced, as well as spontaneous VCO, co-opted vascular cell types (ECs and pericytes) displayed similar transcriptomic signatures to those of their healthy counterparts, distinct from tumor EC (TEC) signatures in angiogenic tumors. Previously reported EC subtypes (136), such as immature, capillary type 1 and 2, artery, vein, and lymphatic ECs, as well as capillary TECs, were found in lung metastases growing *via* angiogenesis, and *via* VCO, as well as in healthy lungs (10). However, capillary ECs expanded in both healthy and co-opted vessels, while angiogenic TECs (tip and proliferating ECs) largely disappeared in co-opted vessels, which may be (at least in part) a reason for the lack of effectiveness of AAT in patients with tumors growing *via* VCO (16, 139). Co-opted pericytes displayed gene signatures of quiescence and vasodilation, similar to those enriched in pericytes from healthy lungs, and distinct from the gene signatures of angiogenic pericytes (**Figure 1**), which involve genes related to activation, vessel sprouting, and matrix remodeling (10). Cancer cells were confirmed to upregulate invasive gene signatures, in line with previous observations (16, 52, 140, 141). Interestingly, in tumors relying on VCO, M1-like, antigen-presenting/inflammatory macrophages, as well as hypoxia-regulated and matrix-remodeling macrophages accumulated, in line with the characteristic hypoxic areas and invasive cancer cells in tumors relying on VCO (10) (**Figure 1**). On the other hand, M2-like, immunosuppressive macrophages were enriched in angiogenic tumors (10) (**Figure 1**). These intriguing observations may implicate potential novel treatment strategies based on macrophage targeting, as discussed above.

While providing important new insights, this study also generated many questions: is there a difference in EC transcriptomes between those in primary tumors or metastases relying on VCO? How do these findings translate into the clinical human setting? Based on the differences on ECs in different organs and vascular beds, are there differences in co-opted vessels, and other co-opted cell types based on location and type of the tumor? How do angiogenic ECs compare to those that are co-opted in (the same) metastatic lesions? Therefore, more single-cell transcriptomic studies, involving different cancer and metastasis models, as well as human samples, are required. Only with a more comprehensive understanding of the biology of ECs and other cell types during VCO, we can move forward to develop novel treatment strategies.

With this in mind, one could consider exploiting already existing single-cell transcriptomic studies, in particular those analyzing metastases, as VCO is thought to be present in a majority of metastatic lesions (16). Several studies have examined cancer cells as well as stromal cells (including vascular cell types) in metastatic lesions (142, 143), which could potentially be re-analyzed and compared to known transcriptome characteristics of co-opted cell types. Although such studies analyzed metastatic lesions at the single-cell level, sub-setting and an in-depth transcriptome analysis of ECs and other vascular cells have not yet been the focus of such studies. In order to gather a more complete picture of angiogenic and co-opted EC biology, one should compare vascular cell transcriptome profiles in primary angiogenic tumors, in angiogenic metastases, and in metastases relying on VCO. While this may be challenging with metastatic lesions relying on VCO, spatial transcriptomic options discussed below may be exploited to address this concern. Ultimately, original studies with confirmed VCO (via histology) are also required to gain full insight to confirm and expand interpretations from re-analyzed studies.

### Where do we go from here?

While single-cell RNA sequencing provides large amounts of transcriptomic information on all cell types in tumors or metastases, spatial information is lost with this approach, preventing the investigation of cell-cell interactions. Nevertheless, several computational approaches, such as CellChat (144) and NicheNet (145), allow prediction of interactions between the different cell types analyzed in a single-cell study. CellChat relies on a database of known receptor-ligand complexes (CellChatDB). The program calculates a probability value of an interaction between two groups of cells, which differently over-express the ligand and the receptor, respectively. NicheNet additionally combines the predicted receptor-ligand interactions with expression data on interacting cells to project the impact of the interactions on the receiver cell’s gene expression, by integrating the anticipated receptor-ligand interactions with intracellular signaling pathways. In fact, CellPhoneDB was used to study interactions between the different cell types identified during VCO at single-cell level (10). Interestingly, different co-opted cell types seem to interact with each other. For instance, M1-like macrophages were predicted to interact with co-opted ECs *via* CXCL12-CXCR4, and oncostatin-M (OSM) receptor-OSM interactions; and with pericytes *via* TGF-β signaling (10). How such interactions may influence the functions and phenotypes of the involved cell types remains to be investigated.

The prediction of interactions of different cell types within a tissue may indeed also allow to speculate spatial relationships between cells. However, considering that the only recognized technique to identify VCO is histological analysis, one cannot omit this method when investigating co-opted vessels. In fact, some sophisticated approaches have been utilized to shed light on mechanisms of VCO. For instance, a 3-dimensional (3D) model of vessel growth in lung adenocarcinoma was established by combining hematoxylin and eosin, immunohistochemistry and multiplex immunofluorescence images of patient samples (146). The authors were able to analyze the spatial relationships of cancer cells with the vasculature and the lung parenchyma, identifying tumor nests in the alveolar spaces lacking CD31-positive vessels, as well as focal attachment of cancer cells (spread through air spaces (“STAS” cancer cells) to the alveolar walls, consistent with VCO. The model proposed that cancer cells can break loose from the tumor bulk, and journey through air spaces to attach to alveolar walls, which are surrounded by capillaries (146).

This 3D modeling strategy demonstrates the importance of spatial information, but on the other hand is limited to probing for a handful of proteins simultaneously. To gather the fine-grained single-cell transcriptomic information yet preserving the positional context of the cells within a tissue, “spatial transcriptomics” was developed. Spatial transcriptomics, which was selected as *Nature Methods’* “Method of the Year” (147), comprises several approaches to perform RNA sequencing on tissue sections, in conjunction with imaging. Examples are fluorescent *in situ* RNA sequencing (FISSEQ) (148), RNA seqFISH+ (149, 150), Slide-seq (151), and the method termed “spatial transcriptomics” (152). The latter technology, which relies on barcoded reverse transcription primers allowing RNA sequencing data to be associated with a precise tissue location, was commercialized and is today known as the 10x Genomics product “Visium”. While spatial transcriptomics does not yet enable analysis at the single-cell level, the continuous progress in the field holds hope for single-cell spatiotemporal analysis in the coming years. In the meanwhile, different computational approaches exist to *in situ* map transcriptome information to spatial information (153–157).

Apart from investigating spatial relationships between cells with known transcriptomes, the VCO field may also benefit from additional novel technologies. For instance, cytometry by time of flight (“CYTOF” (158, 159), an application that uses antibodies conjugated to heavy metal isotopes that can be detected with a mass cytometer), allows simultaneous detection of 40-100 target proteins per cell. Although still not comparable to the vast gene expression information gained by single-cell RNA sequencing, one may argue that protein expression may translate better into cells’ phenotypes and their functions than RNA expression. In line with this, cite-seq is an approach that combines single-cell RNA sequencing with barcoded antibodies, thereby integrating protein and mRNA data (160). Lastly, to gather a comprehensive picture of the VCO process, one should seek to investigate the epigenome of co-opted cell types, to probe for DNA regulatory elements that may be changed in these cell types and provide an additional level of insights into the acquisition of co-opted phenotypes. Techniques, such as single-cell Assay for Transposase-Accessible Chromatin (ATAC) sequencing (161), or Methylome sequencing (162–164) are suitable for such endeavors.

In summary, the new and rapidly evolving era of sequencing and other novel technologies provide seemingly unlimited possibilities to explore VCO on many different levels. Ultimately, these techniques could enable us to understand the VCO process and develop successful therapeutic interventions.

## Concluding remarks

VCO is a well-established, yet at the same time, a mysterious and barely understood process of tumor vascularization. For decades, it has been established that blood supply is vital for tumor growth and angiogenesis as a hallmark of cancer. While several additional vascularization mechanisms have long been recognized, most of them have not received much attention from the scientific community, one of which being VCO. The discovery of VEGF’s role in angiogenesis has led to groundbreaking achievements in cancer therapeutics, with approval of the first anti-angiogenic drug Bevacizumab for cancer in 2004. Unfortunately, although successful in some cancer patients, it became obvious that most cancer patients develop resistance mechanisms and become capable of ensuring their blood supply with alternative strategies. Almost two decades later, no vital tactics have been developed to efficiently counteract such resistance mechanisms. Given the high incidence of VCO in AAT-treated tumors, and in particular in metastases (which in most cancers are the cause of death), this mode of vascularization is of remarkable interest to overcome AAT resistance in tumors. It is thus surprising that VCO has received so little attention. However, with the advent of the single-cell transcriptomic era and the plethora of technologies, many opportunities exist to tackle the unraveling of the still cryptic process of VCO. There is great hope that future studies using novel technologies and analysis methods will illuminate the molecular mechanisms that initiate VCO, as well as the detailed characteristics of co-opted cell types. These insights will then facilitate target identification, and ultimately lead to development of unprecedented treatment strategies for tumors and metastases.

## Author contributions

Conceptualization: PC. Writing manuscript and creating figures: AC, A-CKT, LMB, PS-G. All authors contributed to the article and approved the submitted version.

## Funding

PC is supported by Grants from Methusalem funding (Flemish government), the Fund for Scientific Research-Flanders (FWO-Vlaanderen), the European Research Council ERC Advanced Research Grant EU- ERC74307 and the NNF Laureate Research Grant from Novo Nordisk Foundation (Denmark); LB is supported by a Marie-Sklodowska-Curie Individual Fellowship; AC, A-CKT, LMB are supported by ‘Fonds voor Wetenschappelijk Onderzoek’ (FWO) doctoral and postdoctoral fellowships.

## Conflict of interest

The authors declare that the research was conducted in the absence of any commercial or financial relationships that could be construed as a potential conflict of interest.

## Publisher’s note

All claims expressed in this article are solely those of the authors and do not necessarily represent those of their affiliated organizations, or those of the publisher, the editors and the reviewers. Any product that may be evaluated in this article, or claim that may be made by its manufacturer, is not guaranteed or endorsed by the publisher.

## Abbreviation

Ang-2, angiopoietin-2; AAT, anti-angiogenic therapy; ARP2/3, actin-related protein 2/3 complex; ATAC, assay for Transposase-Accessible Chromatin; B2R, B2 receptor; CA9, carbonic anhydrase 9 CAM chick chorioallantoic membrane; CDC42, cell division control protein 42 homolog; COL4-A3, collagen type IV, α3; CRCLM, colorectal cancer lung metastasis; CXCR4, CXC-chemokine receptor 4; CXCL9, CXC-chemokine ligand 9; CXCL12, CXC-chemokine ligand 12; CYTOF, cytometry by time of flight; ECM, extracellular matrix; ECs, endothelial cells; EGFR, epidermal growth factor receptor; EMT, epithelial-to-mesenchymal transition; ERK, extracellular signal-regulated kinase; FAK, focal adhesion kinase; FISSEQ, fluorescent in situ RNA sequencing; GBM, glioblastoma multiforme; GSK3β, glycogen synthase kinase 3β; HAS1, hyaluronan synthase 1; Hep3B-hCG, human hepatocellular carcinoma; HEVs, high endothelial venules; HGF, hepatocyte growth factor; HGP, histopathological growth pattern; HIF1α, hypoxia-inducible factor 1; IRE1, inositol-requiring enzyme 1; ITGB2, integrin β2; L1CAM, neural cell adhesion molecule L1; LAMA1, laminin α1; LOXL-4, lysyl oxidase like 4; MMP, metalloproteinase; OSM, oncostatin-M; P4HA1, hypoxia enhances prolyl-4-hydroxylase α1; PD-L1, programmed-cell death ligand 1; PD-L2, programmed-cell death ligand 2; PTP1B, protein tyrosine phosphatase 1B; RENCA, renal adenocarcinoma; RUNX1, runt-related transcription factor-1; STAS, spread through air spaces; TAZ, transcriptional co-activator with PDZ-binding motif; TEC, tumor EC; Tie-2, tyrosine kinase-receptor; VCO, tumor vessel co-option; VEGF-A, vascular endothelial growth factor-A; VEGFR2, vascular endothelial growth factor receptor; YAP, Yes-associated protein; ZEB2, Zinc finger E-box binding homeobox 2.
